# Comment on Mahmood, M.N. Direct Immunofluorescence of Skin and Oral Mucosa: Guidelines for Selecting the Optimum Biopsy Site. *Dermatopathology* 2024, *11*, 52–61

**DOI:** 10.3390/dermatopathology12030026

**Published:** 2025-08-26

**Authors:** Şebnem Demiral, Yunus Özcan, Mehmet Gamsızkan

**Affiliations:** 1 Department of Dermatology, Düzce University Faculity of Medicine, Düzce 81060, Türkiye; yunusozcan18@gmail.com; 2 Department of Pathology, Düzce University Faculity of Medicine, Düzce 81060, Türkiye; drgamsiz@yandex.com.tr

**Keywords:** direct immunofluorescence, vesiculobullous disorders, immunopathology

Compilation written by Muhammad N. Mahmood [1] is quite educational and a useful resource for clinicians. Direct immune fluorescence technique is a histopathological diagnostic method that visualizes antigen–antibody interactions with fluorescently labeled antibodies and is used to diagnose vesiculobullous disorders, vasculitic, and connective tissue diseases. However, preanalytical factors that depend on the clinician, such as poor biopsy site selection or unsuitable transport media, may lead to pitfalls. Increasing clinicopathological correlation will make it easier to interpret DIF results, boosting the accuracy. To aid clinicians, we prepared a chart depicting the appropriate biopsy sites based on differential diagnosis.

DIF results are highly reliable in differentiating the final diagnosis in most cases. To enhance diagnostic accuracy, the optimal biopsy site must be selected. The pathology request form is an essential component of clinicopathological correlation that promotes open communication. Clinical photos of the sampling place may aid in the diagnostic process. Patient’s topical and systemic medications should be questioned because they may have an impact on pathological examinations.

Pemphigus vulgaris and bullous pemphigoid are two major forms of vesiculobullous disorders. While both cause significant morbidity, pemphigus vulgaris still displays high mortality rates worldwide, necessitating early diagnosis. Pemphigus vulgaris typically begins with oral mucosal involvement followed by Nikolsky-positive flaccid bullae. Bullous pemphigoid is widespread in older people with an onset of urticarial plaques followed by tense bullae. IgA pemphigus is pruritic and mostly affects axillae and groin, causing pustules in addition to bullae, sometimes with a central clearing pattern. Paraneoplastic pemphigus is frequently associated with hematological malignancies such as non-Hodgkin lymphoma, chronic lymphocytic leukemia, and Castleman disease. It is characterized by significant involvement of the oral and ocular mucosa and displays combined features of pemphigus vulgaris and erythema multiforme, both clinically and histopathologically.

Dermatitis herpetiformis (DH) is a cutaneous symptom of celiac disease that causes an itching and blistering rash, usually on the elbows, knees, and buttocks. The shared pathophysiology of celiac disease and DH is antibodies against tissue transglutaminase detected in serum and the small intestine. DH often develops in adulthood and is more common among males than females. In DH, DIF investigation reveals granular IgA accumulation in the papillary dermis.

Cutaneous porphyria is a group of diseases characterized by skin lesions caused by UV sensitivity. Porphyria cutanea tarda is the most prevalent human porphyria, caused by hepatic uroporphyrinogen decarboxylase deficiency, which is acquired in the presence of iron excess. Porphyria is caused by various susceptibility factors, including alcohol misuse, smoking, hepatitis C virus infection, HIV infection, iron overload, and HFE gene mutations. UV exposure causes burning pain, redness, swelling, and blisters on the skin with porphyria. Typically, the hands, arms, and face are affected. Patients have fragile, thin skin. Itching may also occur. Porphyria may manifest as red or brown urine.

A genetic condition known as hereditary epidermolysis bullosa is characterized by mucocutaneous fragility and results in skin blisters from minimal damage.

Skin manifestations are frequently present in vasculitis, sometimes as the first sign. Clinical presentation may differ by the affected vessel size. Small-vessel vasculitis is characterized by palpable purpura, while nodules, ulcers, and livedo reticularis are linked to medium-vessel vasculitis. Large-vessel vasculitis may cause cutaneous necrosis in the perfused area. In contrast, capillaritis is characterized by non-palpable petechiae and purpura, with varying clinical appearances. However, they share similar pathological findings such as endothelial edema, dilated vessels, erythrocyte extravasation, hemosiderin deposition, and perivascular lymphocytic–macrophage infiltration. In contrast, they show no vasculitic changes and are negative or nonspecific for immunofluorescence tests [2].

Discoid lupus erythematosus (DLE) is a persistent disorder that causes scarring and dispigmentation on the skin. This is the most prevalent type of cutaneous lupus. It is distinguished by persistent scaly plaques on the scalp, face, and ears, which can eventually lead to scarring, atrophy, dyspigmentation, and irreversible hair loss in affected hair-bearing areas. DLE can occur at any age, but most frequently occurs in females aged around 20–40. It may be localized or generalized. DLE is characterized by round, coin-shaped, indurated lesions more commonly localized on the scalp and face. They may be red, thick, or scaly. Scars or discoloration of the skin may remain after the lesions disappear.

As a result, based on the information in your article, we have tabulated the locations where a biopsy can be performed on the images of the patients who were admitted to our clinic (Figure 1). The visual and figure material we suggest can help clinicians better understand this concept.

## Figures and Tables

**Figure 1:**
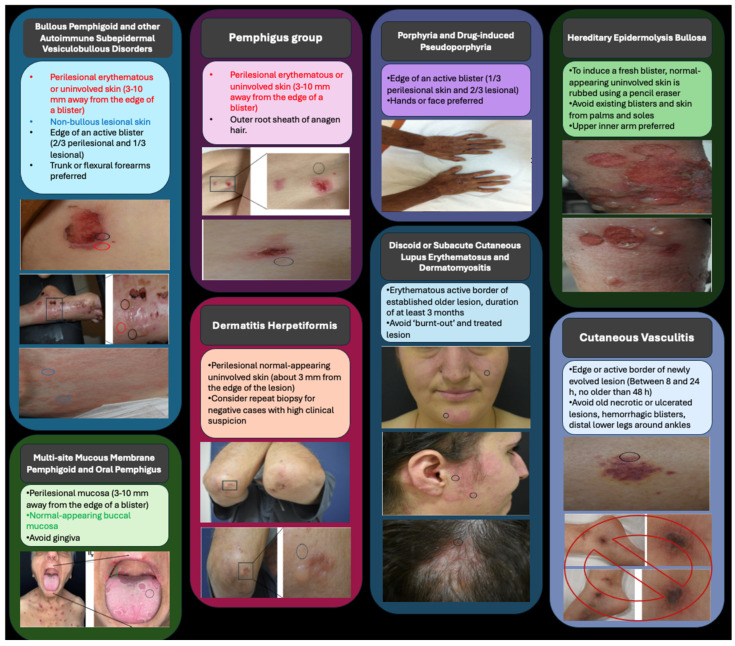
Choosing the optimal biopsy site for direct immunofluorescence studies in various dermatological diseases.

## Data Availability

This article did not incorporate any new data beyond the photographs of patients.
